# Granulomatous Polyangiitis With Renal Involvement: A Case Report and Review of Literature

**DOI:** 10.7759/cureus.19814

**Published:** 2021-11-22

**Authors:** Thoyaja Koritala, Tuoyo O Mene-Afejuku, Matthew Schaefer, Lavanya Dondapati, Yelena Pleshkova, Farah Yasmin, Hisham Ahmed Mushtaq, Anwar Khedr, Ramesh Adhikari, Abbas Al Mutair, Saad Alhumaid, Ali A Rabaan, Jaffar A Al-Tawfiq, Nitesh K Jain, Syed Anjum Khan, Rahul Kashyap, Salim Surani

**Affiliations:** 1 Hospital Medicine, Mayo Clinic, Mankato, USA; 2 Radiology, Mayo Clinic, Mankato, USA; 3 Internal Medicine, Dr. N.T.R University of Health Sciences, Vijayawada, IND; 4 Clinical Sciences, University of Houston, Houston, USA; 5 Internal Medicine, Dow University of Health Sciences, Karachi, PAK; 6 Critical Care Medicine, Mayo Clinic, Mankato, USA; 7 Medicine, Tanta University Faculty of Medicine, Tanta, EGY; 8 Hospital Medicine, Franciscan Health, Lafayette, USA; 9 Geriatrics, Brown University, Providence, USA; 10 Emergency Medicine, Almoosa Specialist Hospital, Al-Ahsa, SAU; 11 Pharmaceutical Care, Al-Ahsa Health Cluster, Al-Ahsa, SAU; 12 Molecular Microbiology, Johns Hopkins Aramco Healthcare, Dhahran, SAU; 13 Infectious Diseases, John Hopkins Aramco Health care, Dhahran, SAU; 14 Anesthesiology and Critical Care, Mayo Clinic, Rochester, USA; 15 Anesthesiology, Mayo Clinic, Rochester, USA; 16 Medicine, Texas A&M University, College Station, USA; 17 Medicine, University of North Texas Dallas, Dallas, USA; 18 Internal Medicine, Pulmonary Associates of Corpus Christi, Corpus Christi, USA; 19 Clinical Medicine, University of Houston, Houston, USA

**Keywords:** wegener’s granulomatosis, wheezing, weight loss, pulmonary cavitation, hemoptysis, chronic sinusitis

## Abstract

Granulomatosis with polyangiitis (GPA), formerly named Wegner’s granulomatosis is an antineutrophilic cytoplasmic antibody (ANCA) associated vasculitis of the small vessels. GPA can affect several organ systems even though predominantly affects respiratory and renal systems. Pathogenesis is initiated by activation of the immune system to produce ANCA, Cytoplasmic (C-ANCA) antibody, which thereby leads to widespread necrosis and granulomatous inflammation. Multisystem involvement with varied symptomatology makes GPA diagnosis more challenging. Early diagnosis and management are vital and can alter the prognosis of the disease. We present a literature review and a clinical scenario of a 26-year-old male with a history of chronic sinusitis, testicular carcinoma in remission, recent onset of worsening cough, epistaxis, hoarseness of voice, weight loss, and dark-colored urine. Workup revealed high titers of C-ANCA, C-reactive protein, procalcitonin, CT chest evidence of mass-like consolidation, and bronchoscopy findings of friable tissue that was not amenable for biopsy. Methylprednisolone and rituximab (RTX) were administered, which resulted in marked clinical improvement. Therefore, a keen eye for details is necessary to diagnose GPA early, which can improve disease outcomes dramatically.

## Introduction

The three subtypes of antineutrophil cytoplasmic antibody (ANCA) associated vasculitides are microscopic polyangiitis (MPA), granulomatosis with polyangiitis (GPA), and eosinophilic granulomatosis with polyangiitis (EGPA). Among the three, GPA is the most common [1]. GPA, previously named Wegener's granulomatosis, is a small-medium vessel disease characterized by necrotizing and granulomatous vasculitis that affects predominantly the respiratory tract as well as the kidney [1]. GPA comprises a triad of (a) upper respiratory tract (sinusitis, crusting rhinitis, saddle nose deformity, mastoiditis, hearing loss, otitis media) and lower respiratory tract (lung nodules, alveolar hemorrhage), (b) systemic (pauci-immune) vasculitis, and (c) renal involvement (glomerulonephritis) [2]. The global incidence of GPA is estimated to be 10-20 cases per one million annually, depending upon the geographical location [2]. A higher incidence is noted in the colder climatic areas [2,3]. The incidence rate in the United States is 12.8 cases/million person-years in working-age adults and 1.8 cases/million person-years in children [3]. GPA is generally seen in the geriatric population with a peak at 64-75 years of age, and recent studies demonstrated no gender preference or predisposition [4]. Even though the peak incidence is among older individuals, it may still present in the younger individuals and therefore posing a great diagnostic challenge as it was in this case presentation. GPA is commonly documented in Whites, although it is noted in all racial and ethnic groups [2].

We report an interesting case of a young man with multiple chronic medical problems who presented to the ER with life-threatening symptoms in whom a high index of suspicion triggered an early diagnosis of GPA, resulting in a prompt therapy leading to excellent clinical outcomes.

The diagnosis of GPA is very intriguing because it mimics many other disease processes. Undiagnosed GPA may have devastating consequences. Prompt diagnosis and therapy, on the other hand, may alter the grim prognosis of this disease which is regarded as a great masquerader. In fact, early therapy has been associated with survival rates as high as 83% in year one and 74% at five years [5,6].

## Case presentation

A 26-year-old male with a history of chronic sinusitis status post-surgery for nasal polyps and deviated septum (five years prior to presentation); prior nicotine dependence, psoriasis, and testicular cancer status post-surgery and chemotherapy (two years prior to presentation) currently in remission presented to the hospital because of acute worsening of chronic cough and shortness of breath. His presentation to our hospital's ER was preceded by a clinic visit for chronic sinusitis, where he was prescribed a 10-day course of oral amoxicillin/clavulanate at that time. He had also been previously evaluated by the otorhinolaryngologist a few months back, who did a CT scan of the sinuses that revealed chronic pansinusitis. He was being prepped for more surgeries (debridement) for the sinus problems when he had progressive worsening of shortness of breath.

At the time he presented to our ER, he was short of breath at rest and had an audible wheeze. He had a cough productive of blood-tinged sputum, pleuritic chest pain, and right-sided neck pain. He had associated night sweats and weight loss (lost approximately 25 lbs. in the last two months). Positive history of chronic nasal congestion, impaired hearing, and epistaxis was noted. He also had new-onset hoarseness of voice and passage of dark-colored urine. He denied contact with persons with chronic cough or residence in areas noted to be endemic for tuberculosis. He was using adalimumab once monthly for plaque psoriasis of the knee, with the last dose taken a month prior to the presentation. He denied having nausea, vomiting, diarrhea, rashes, lower extremity swelling, orthopnea, or dysuria. Vitals were significant for temperature 37.1°C, pulse 108/min, respiratory rate 20 /min, blood pressure 128/79 mmHg, and oxygen saturation at 88% on room air, which improved to 98% after the administration of two liters of oxygen via nasal cannula.

**Figure 1:**
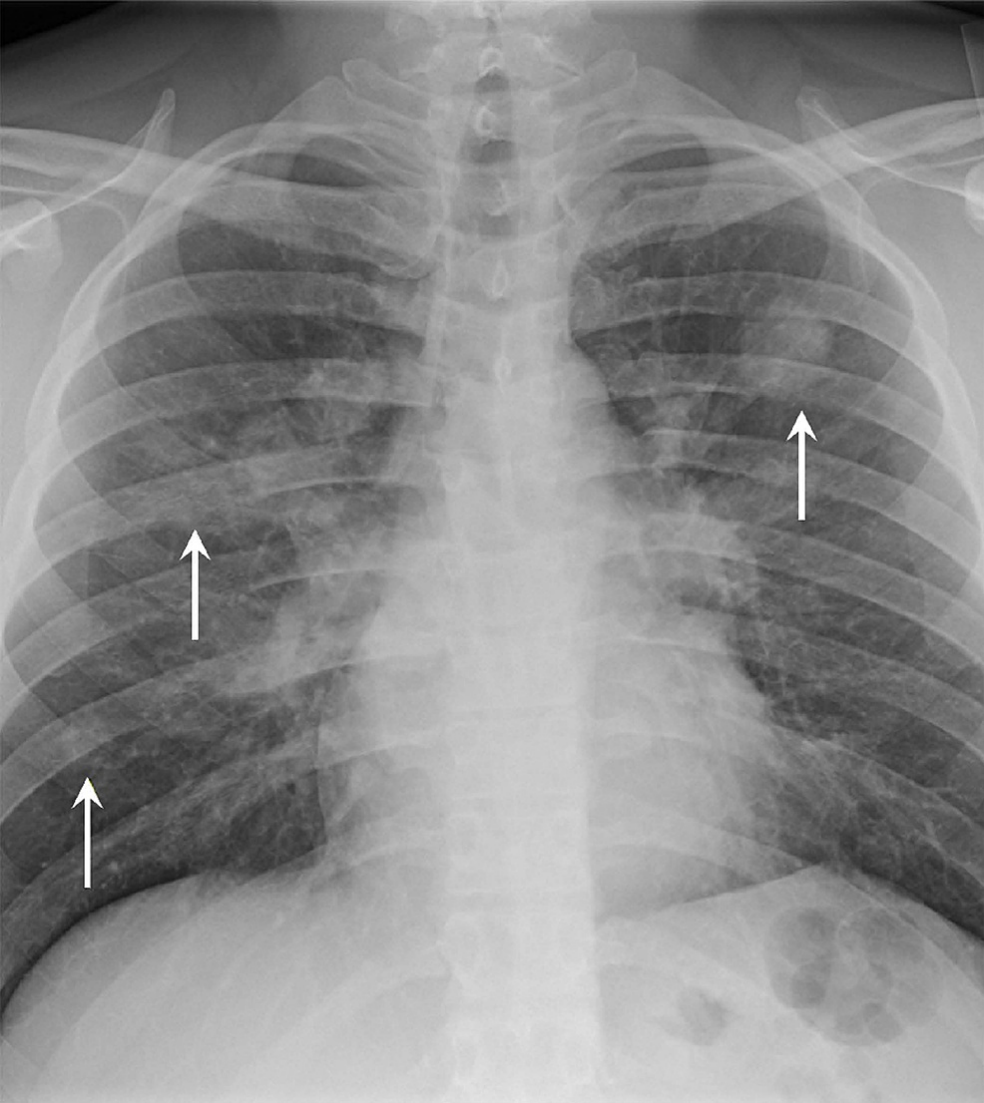
A chest radiograph anteroposterior view showing multifocal bilateral nodular airspace opacities (arrows).

Laboratory workups were notable for worsening normocytic anemia with hemoglobin of 9.1 g/dl, white blood cell count of 14,100/µL with neutrophil predominance, platelet count elevated to 639,000/µL, C-reactive protein (CRP) 242 mg/L (reference range, <=8 mg/L), albumin 3.2 g/dl, procalcitonin 0.18 ng/ml (reference range, <=0.08 ng/ml), and hemoglobin A1c 6.0%. Urinalysis revealed blood, dysmorphic red cells, and protein. Infectious workup was negative for bacteremia, methicillin-resistant *Staphylococcus aureus* nasal screen, legionella, and *Streptococcus pneumonia* urine antigens, serology of Aspergillus, Blastomyces, and Cryptococcus, *Pneumocystis jiroveci *pneumonia, *Mycobacterium tuberculosis*, HIV, influenza A/B, respiratory syncytial virus, and COVID-19 infections. He had a chest x-ray that revealed multifocal bilateral nodular appearing airspace opacities (Figure 1).

**Figure 2:**
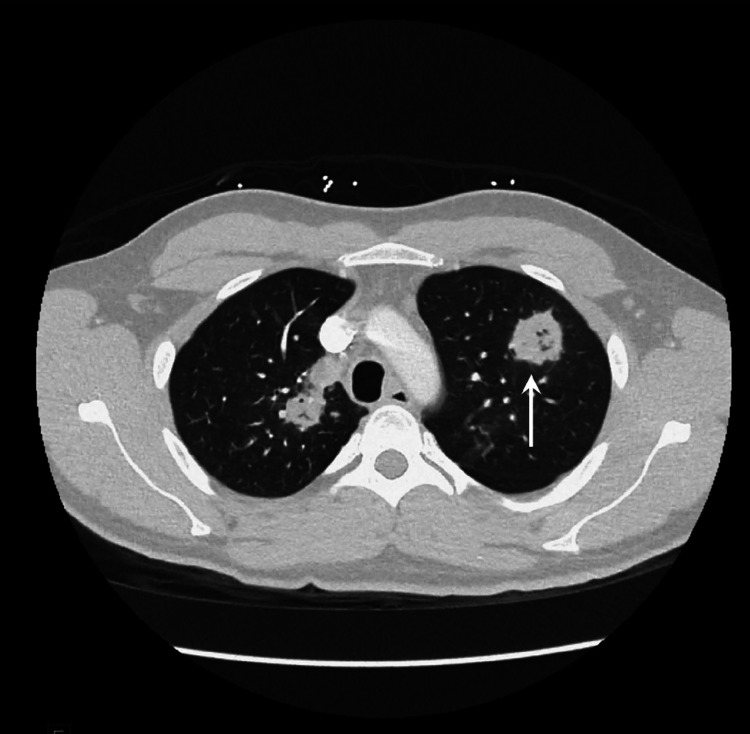
Chest CTA showing scattered areas of mass-like consolidation and areas of early cavitation (arrow).

He also had a chest CT angiogram for better characterization of the lesions seen on the chest x-ray. The chest CT (Figure 2) revealed scattered areas of mass-like consolidation in a predominantly peri-hilar and peripheral distribution within the lungs. Some areas appeared to have early cavitation.

**Figure 3:**
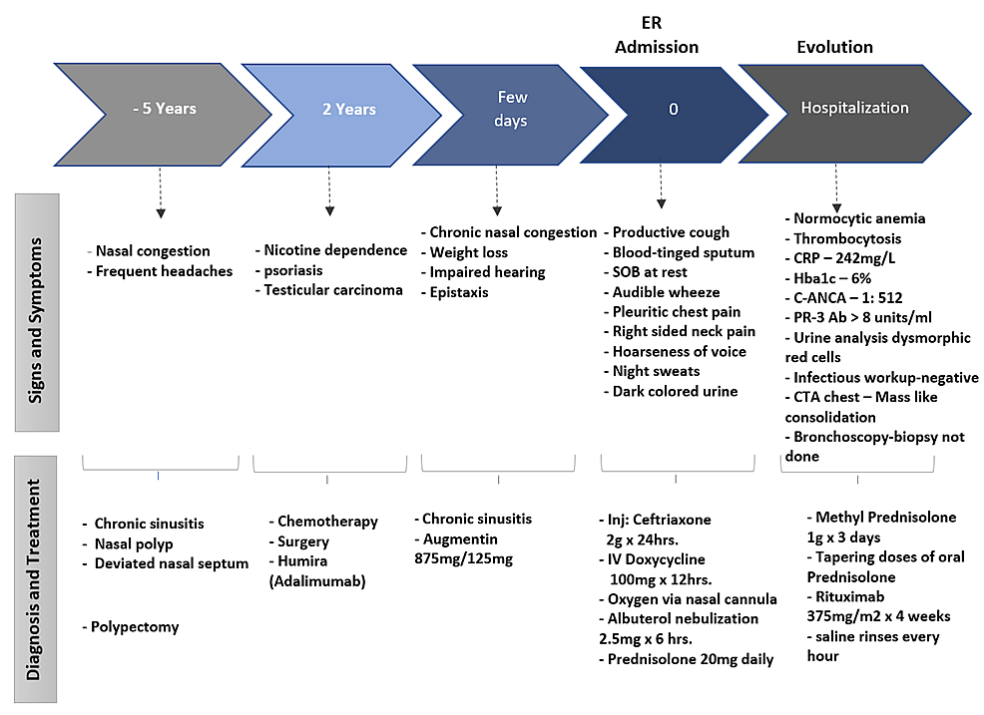
Timeline of the evolution of GPA and management in our patient.

Vasculitis was considered as the top differential because of the constellation of chronic sinusitis, normocytic normochromic anemia, thrombocytosis, cavitary lung lesions, hemoptysis, asthma-like symptoms, hoarse voice, hematuria, and hearing loss. The results of the antinuclear antibody test (ANA) came back negative, but C-ANCA was positive 1:512 (reference range: negative), and proteinase three antibodies > 8 units/ml (reference range <0.4units/ml) were elevated. He underwent bronchoscopy to confirm the diagnosis of GPA and to further evaluate for any infection. Biopsy was not obtained during bronchoscopy due to the friability of the tissue. Nephrology did not recommend kidney biopsy, given urinalysis being consistent with kidney involvement. He was started on pulse dose steroids of 1g methylprednisolone for three days with subsequent tapering doses of oral prednisone along with *Pneumocystis jiroveci* prophylaxis with trimethoprim-sulfamethoxazole and calcium and vitamin D supplementation. He continued saline nasal rinses every hour as recommended by an otorhinolaryngologist. He was also started on rituximab (RTX) 375 mg per m2 per week for four weeks after negative chronic hepatitis panel results were obtained. Figure 3 details the timeline of events and management in our patient.

He was recommended follow-ups with vasculitis clinic and otorhinolaryngology for subglottic stenosis in four to six weeks in the outpatient setting. Preventative vaccinations for COVID-19 upon discharge from the hospital were recommended due to the initiation of RTX. He was also advised to get the varicella vaccine (shingles), human papillomavirus vaccine (HPV), and pneumococcal vaccine. He had routine clinic follow-up as well as repeat bronchoscopies (two and three months after the index bronchoscopy), which revealed that the tracheal inflammation had much improved. The patient has been stable ever since.

## Discussion

GPA is primarily characterized by a triad of necrotizing granulomatous inflammation of the upper and/or lower respiratory tract, necrotizing glomerulonephritis, and an autoimmune necrotizing systemic vasculitis affecting predominantly small vessels [7]. GPA primarily involves both the upper and lower respiratory tract and kidney. It can affect any organ in the body [8,9]. Infections like hepatitis B and C, drugs like hydralazine, and neoplasms are known to be associated with vasculitis. The pathogenesis of the disease is complex, ranging from an indolent disease involving only one site to fulminant, multiorgan vasculitis leading to death [8-10]. Most of the hallmark symptoms of the disease may be lacking initially but may evolve later. Cardinal histologic features on biopsy show widespread "geographic" necrosis and granulomatous inflammation [10]. ANCAs play a major role in the pathogenesis and often are associated with the activity of the disease [8-10]. The presence of ANCAs targeted against proteinase 3 (PR3-ANCA) is highly specific for Wegener's granulomatosis. myeloperoxidase (MPO)-ANCA or negative ANCA is seen in EGPA (Churg-Strauss syndrome) and MPA, which are the other two ANCA-associated vasculitides [11]. On biopsy, Wegener's granulomatosis can be distinguished from its counterparts when the inflammatory infiltrates have a granulomatous pattern [12]. ANCA is detected only in approximately 50% of the patients with localized GPA (which is limited to the respiratory tract and affects about 5% of the patients), whereas PR3-ANCA antibodies are detected in 95% of the patients with generalized GPA [10-13].

Clinical features

Clinical manifestations of GPA are protean; GPA can affect almost any organ. 

Upper Airway Manifestations

More than 85% of the patients with GPA often present with upper respiratory tract symptoms that include chronic sinusitis, epistaxis, or otitis media [7,8,14-16] with abnormal findings in sinus CT scan, as seen in our patient [7,12]. Typical features include destruction of sinus bones (25-50%) and thickening or clouding of the sinuses (75%) [7,12]. Ear involvement presents in 30-50% of patients with GPA [7,12] and 20-30% of the patients will have hearing loss, otitis media, otalgia, chronic mastoiditis [7,8,12]. The nasopharynx is affected in 60-80% of the patients with GPA [7,8,15]. Nasal congestion, bleeding, ulcers, septal perforation [7,16,17], and saddle nose deformity [7,8,15] may occur. Also, hoarseness or sore throat may reflect ulcerations or granulomatous involvement of the vocal cords or pharynx, as seen in our patient during his acute presentation for worsening shortness of breath [7]. 

Pulmonary Manifestations

GPA is the most common pulmonary vasculitides [12]. It most commonly affects lung parenchyma characterized by multiple nodules and masses, which is seen in 55-90% of GPA [2,6,18,19]. Symptoms include cough, dyspnea, impaired pulmonary function, bronchial stenosis, abnormalities on chest radiographs or CT scans, and diffuse alveolar hemorrhage (DAH) [4,6,18]. Chest imaging reveals lesions in 70% or more patients [7,19,20]. Characteristic findings are solitary or multiple nodules, cavitation, or infiltrates, as seen in our case presentation [7,19,20]. Other features of GPA include pleural effusions, tracheobronchial stenosis, septal bands, atelectasis, mass lesions, asymmetrical pleural thickening, and scarring [21-24]. Surgical lung biopsy (SLB) is ideal for diagnosing pulmonary GPA, which mostly reveals vasculitis and necrotizing granulomas [25,26]. Travis et al. [25] studied 87 open lung biopsies from 67 patients with pulmonary GPA, which reports the findings of vascular inflammation in 96%, parenchymal necrosis in 84%, dispersed giant cells in 79%, areas of geographic necrosis in 66%, granulomatous microabscesses with giant cells in 70%, and neutrophilic microabscesses in 65% of patients. Additionally, active GPA consists of focal infiltrate, consolidation, or ground-glass opacities [20-22]. Consolidation may reflect alveolar hemorrhage or granulomatous inflammation [27,28]. DAH secondary to a small vessel vasculitis may be the initial and only pulmonary manifestation of GPA [23].

Renal Manifestations

Glomerulonephritis (GN) is the most common renal pathology affecting 70 to 85% of GPA, but renal inadequacy considered as serum creatinine > 2.0 mg/dL involves only 10-15% of patients at presentation [6-8]. Usually, red cell casts, proteinuria, microscopic hematuria are seen prior to an increase in serum creatinine levels [6,7,12]. The typical renal lesion of GPA is a focal segmental GN without immune complexes, “pauci-immune GN.” [7,12]. Sometimes crescentic rapidly progressive glomerulonephritis (RPGN) is observed with the fulminant disease [12,27,28]. Granulomatous vasculitis is found in only 6-15% of renal GPA [6,7,12,28]. The disease onset and progression of renal involvement is variable, ranging from indolent (months to years) to fulminant or progressing to end-stage renal disease (ESRD) within days to weeks [27,28]. Dialysis-dependent ESRD develops in 10-33% of patients with GPA [7,12,28]. Extensive loss of glomeruli with crescentic or sclerotic features is seen in chronic dialysis-dependent renal failure biopsy specimens [27,28]. 

In a prospective study done by Andrassy et al. [29] to assess the survival and prognosis of 25 patients with biopsy-proven renal GPA, 14 patients ended up requiring dialysis at admission, four of whom went on to develop ESRD. Another prospective study by Gottenberg et al. [30] assessed the long-term outcome and prognosis among 37 patients with GPA and renal disease. During a period of 6.4 years, 15 out of 37 (41%) died, and two developed ESRD [30]. Similarly, Fauci et al. [6] conducted a prospective study in treating and closely studying 85 patients with well-documented Wegener's granulomatosis over a 21-year period who were treated with chronic low-dose cyclophosphamide together with alternate-day corticosteroid. Seventy-two patients (85%) had well-documented renal disease. Results of percutaneous renal biopsy in 58 of these 72 patients showed renal disease, whereas the remaining 14 patients had urinary sediment or functional abnormalities [6]. The renal histopathology ranged from mild focal and segmental glomerulonephritis with minimal urinary findings to fulminant diffuse necrotizing glomerulonephritis with proliferative and crescentic changes [6]. Extrarenal manifestations consistently precede renal disease in Wegener's granulomatosis [6]. However, renal disease, once present, may progress from mild to severe glomerulonephritis within weeks and even days of its inception [6].

Trachea and Bronchi Manifestations

About 10-30% of patients with GPA develop tracheal or bronchial stenosis [7,12,31], where simultaneous involvement of the nasopharynx or sinuses is also seen [32]. Usually, 3-5 cm infra glottic region is affected, but a distal tracheobronchial tree can also be involved, which can occur even in mild disease [31-33]. Stenosis can be detected on bronchoscopy in the form of circumferential narrowing [32]. Biopsy findings of the trachea or bronchi are usually nonspecific [32,33]. Thin-section CT helps in the assessment of the depth of tissue spread [34-36]. MRI and laryngoscopy provide comparable results for grading of subglottic stenosis in GPA and correlate well with pulmonary function testing [36].

Ocular Manifestations

Ocular symptoms are seen in 20-50% of patients with GPA [7,8,37]. Pathogenesis involves the spread of the granulomatous inflammation from the adjacent soft tissues or sinuses into the orbit or due to primary vasculitis of the retinal blood vessels [38]. About 15-20% of patients with GPA develop conjunctivitis, episcleritis, and scleritis [6,39]. Rare complications are optic neuritis, necrotizing keratitis, posterior uveitis, corneoscleral perforation [39]. Corticosteroids are the mainstay of treatment, topical or systemic, depending on the severity. Immunosuppressive therapy is added for severe disease [6,39]. The involvement of optic chiasm or orbit is treated by surgery [38,40].

Nervous System Manifestations

Nervous system spread, central or peripheral, is seen in less than 5% of GPA but can increase to 15-55% as the disease progresses [7,12,41]. Pathogenesis is related to vasculitis of the nervous tissues per se or contiguous spread of inflammation or granulomatous pathology affecting the spinal cord, meninges, and brain [42,43]. Most common are mononeuritis multiplex or polyneuritis comprising more than 50% pathology [42,43]. Other pathologies include stroke, cranial nerve palsies [42,43], diabetes insipidus (secondary to granulomatous involvement of the hypothalamus), focal deficits or seizures, altered mental status, or cognitive impairment, and pituitary insufficiency [44-46].

Joint Manifestations

Musculoskeletal pathology occurs in 30-50% of the patients manifesting as arthralgia, arthritis, and myalgia [47]. Knee and ankles are more commonly affected [48]. Often musculoskeletal GPA can be misdiagnosed as rheumatoid arthritis (RA), where cyclic citrullinated peptide (CCP) antibody is measured to differentiate both the diseases [49].

Cardiac Manifestations

Cardiac involvement is rare, but prevalence rates of 3.3-15% have been estimated [50]. Pericarditis and coronary arteritis are the most common pathology [51]. Complications include heart failure, arrhythmias, or cardiomyopathy [51,52]. Echocardiography, especially transesophageal echocardiography, can easily identify and delineate cardiac, proximal aortic involvement and may also be used to assess response to treatment [50].

Treatment

New concepts are evolving to treat GPA. Corticosteroids and cyclophosphamide (CYC) have been the cornerstone for the management of generalized, multisystemic GPA since the 1970s [6]. Later, recent advances in the treatment proved the efficacy of RTX, a monoclonal antibody directed against B cells. Moreover, RTX use is beneficial for avoiding the side effects caused by CYC, inducing remission, and decreasing relapse rates [12]. This was the mainstay of therapy in our patient, and this appeared to have been associated with good clinical response.

RTX, methotrexate, mycophenolate, and azathioprine are used to maintain remission of GPA. For patients with GPA without renal involvement, corticosteroids along with weekly oral methotrexate of 20-25 mg are utilized instead of RTX or CYC [53]. Mycophenolate mofetil is used for treating patients who have GPA relapses or inadequate response despite receiving therapy with CYC and/or RTX. Patients who are intolerant of CYC or RTX can be treated with mycophenolate, azathioprine, or methotrexate [54]. In patients with life-threatening illnesses, concurrent therapy with RTX and CYC is initiated to circumvent the poor prognosis. Patients on high-dose corticosteroid therapy should have pneumocystis prophylaxis, blood glucose monitoring, bone density assessment, and supplemental vitamin D, and calcium as needed. Since infection in the first year of therapy is associated with mortality and morbidity, annual influenza and pneumococcal vaccines are recommended, along with tuberculosis and hepatitis B screening. Given the multisystem involvement, our patient was treated with methylprednisolone and RTX with the intent of inducing remission. As he was discharged on prolonged steroid taper, pneumocystis prophylaxis with trimethoprim-sulfamethoxazole, insulin for glycemic control, calcium, and vitamin D supplements were recommended along with COVID-19, varicella, pneumococcal, and HPV vaccines. Multidisciplinary care, as in our patient, is warranted in the outpatient setting to ensure optimal recovery and maintenance of remission. 

## Conclusions

GPA very frequently can be missed due to its varied and misleading symptomatology, contributing to delayed treatment often leading to poor clinical outcomes. GPA should be considered as a diagnosis when the presentation involves multiple systems, including joints, kidneys, and lungs. In cases where patients present with atypical symptoms and signs, it would be beneficial to include immunological testing such as the ANCA test to accurately diagnose GPA. Early diagnosis of GPA is very crucial for providing the right treatment in such cases. Lifesaving treatment of GPA is based on prompt and aggressive administration of pulse steroids, and RTX may improve outcomes.
